# An Intranasal Ectopic Tooth in an Adult

**DOI:** 10.7759/cureus.24410

**Published:** 2022-04-23

**Authors:** Radvile Raubaite, Akvile Rakauskaite, Donata Sukyte-Raube, Linas Zaleckas, Darius Rauba

**Affiliations:** 1 Institute of Odontology, Faculty of Medicine, Vilnius University, Vilnius, LTU; 2 Faculty of Medicine, Vilnius University, Vilnius, LTU; 3 Center of Ear, Nose and Throat Diseases, Vilnius University Hospital Santaros Clinics, Vilnius, LTU; 4 Centre of Oral and Maxillofacial Surgery, Institute of Odontology, Faculty of Medicine, Vilnius University, Vilnius, LTU; 5 Centre of Oral and Maxillofacial Surgery, Vilnius University Hospital Zalgiris Clinic, Vilnius, LTU; 6 Clinic of Ear, Nose, Throat and Eye Diseases, Faculty of Medicine, Vilnius University, Vilnius, LTU

**Keywords:** paranasal ct, nasal obstruction, supernumerary tooth, nasal tooth, intranasal ectopic tooth

## Abstract

Ectopic teeth can be supernumerary, deciduous or permanent, and can occur in a wide variety of locations outside of the cavity of the mouth. While supernumerary teeth are rare, supernumerary intranasal teeth are rarer. It is not clear what causes the eruption of teeth intranasally; however, trauma, infection, radiation, and developmental defects may be significant factors in their etiology. We report the case of a 33-year-old woman who presented in the otorhinolaryngology department with complaints of rhinorrhea, nasal obstruction, snoring, pain in the forehead, and bad odor that did not improve with conservative treatment. She had a history of extraction of a supernumerary tooth located in the hard palate. During the endoscopic examination, a second tooth-like body was found in the right nasal cavity, which was later surgically removed with endoscopic guidance. During the follow-up visits at three, six, and 12 months, the patient showed a significant reduction of symptoms with remaining rare reoccurrence of mild sinusitis more prominent on the left side as seen in CT scan, thus presumably unrelated to the ectopic intranasal tooth. Although an intranasal ectopic tooth is a very rare finding, it may cause significant morbidity and its removal improves the quality of life of the patient. The benefits of endoscopic removal are greater visibility, better illumination, and precision in surgical removal.

## Introduction

Ectopic teeth can be supernumerary, deciduous or permanent, and can occur in a wide variety of locations outside of the cavity of the mouth. Supernumerary teeth generally affect 0.1-1% of the general population [1,2]. There is no clear etiological cause for the development of intranasal ectopic teeth; however, associations with maxillofacial trauma, odontogenic or rhinogenic infections, developmental defects, and hereditary factors have been reported [2]. Clinical manifestations can be very diverse and can include external deformity, obstruction of the nose, deviated septum, nasal abscess, epistaxis, pansinusitis, oronasal fistula, facial pain, and persistent mild fever [3-5]. Surgical extraction of the tooth may be beneficial for reducing symptoms and preventing potential complications. In this article, we present a case of an intranasal ectopic tooth removed with endoscopic guidance.

## Case presentation

**Figure 1:**
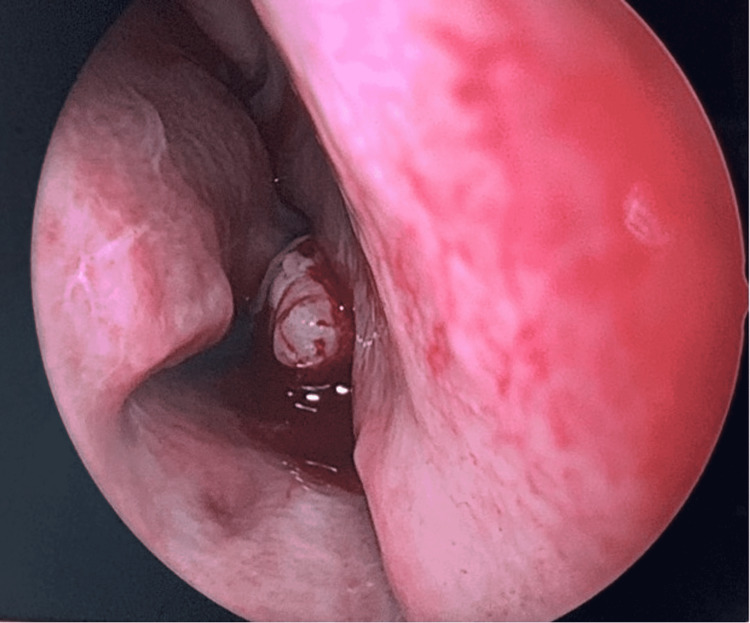
Endoscopic examination of the nose showing the body on the floor of the right nasal cavity.

A 33-year-old woman was referred to an otorhinolaryngologist. She complained of rhinorrhea, nasal obstruction, snoring, bad odor, and pain in the forehead that did not improve with conservative treatment. The patient could not distinguish the time when the symptoms began; however, she pointed out that rhinorrhoea and bad odor intensified in the winter. For the relief of the symptoms, the patient tried using a variety of nasal sprays and was prescribed antibiotics, though the treatment did not work. According to the patient, she had undergone extraction of a supernumerary tooth located in the hard palate during adolescence. Upon otorhinolaryngological examination, a hard, immobile, white-colored, tooth-like body was observed on the floor of the right nasal cavity. The tip of the body was surrounded by nasal mucosa, covered in fibrin (Figure 1). An unpleasant odor was felt when moving the tooth by an instrument. The nasal septum was deviated to the right.

**Figure 2:**
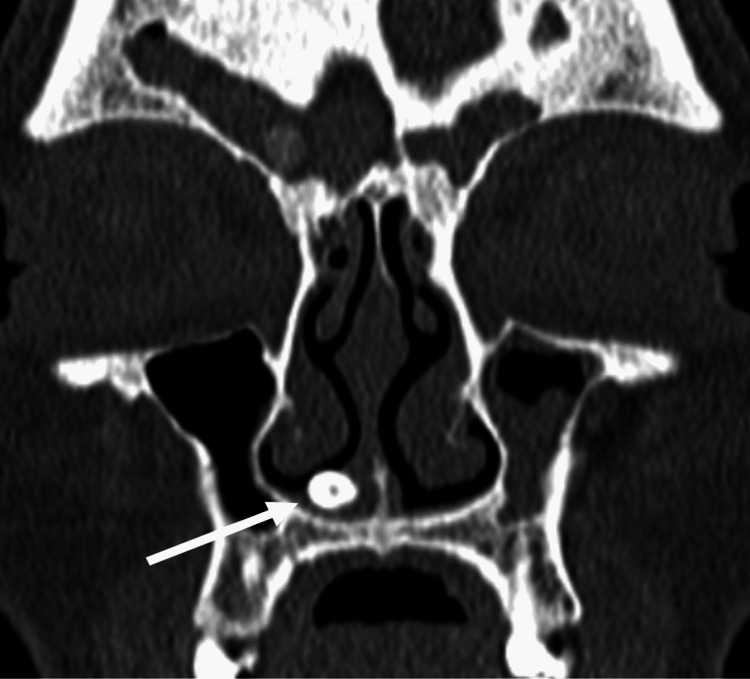
Preoperative coronal computed tomography imaging of the paranasal sinuses showing an intranasal tooth (white arrow) with a focal central radiolucency (resembling dental pulp) arising into the right nasal cavity.

**Figure 3:**
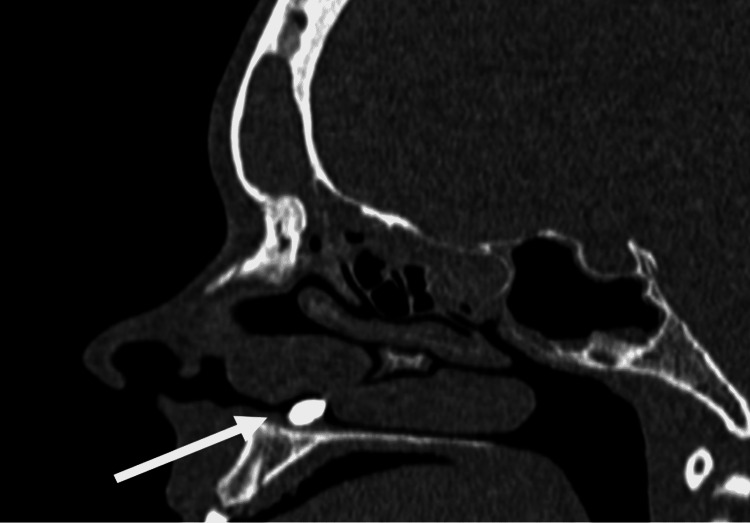
Preoperative sagittal computed tomography imaging of the paranasal sinuses showing an intranasal tooth (white arrow).

**Figure 4:**
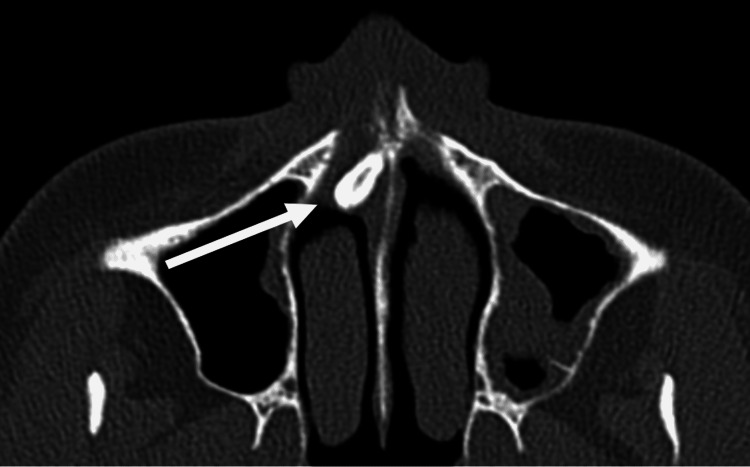
Preoperative axial computed tomography imaging of the paranasal sinuses showing an intranasal tooth (white arrow).

A paranasal sinus CT scan in the axial, coronal, and sagittal planes showed a 12 mm in length and 6 mm in width smooth mass of a homogeneous bone density with tooth-like focal radiolucency arising from the right nasal cavity floor (Figure 2. Figure 3, Figure 4). Additionally, thickened mucosa was observed in the left frontal sinus, ethmoidal sinus, the right maxillary sinus, and the sphenoid sinus suggesting pansinusitis, more prominent on the left side, consequently most likely not related to the ectopic intranasal tooth.

**Figure 5:**
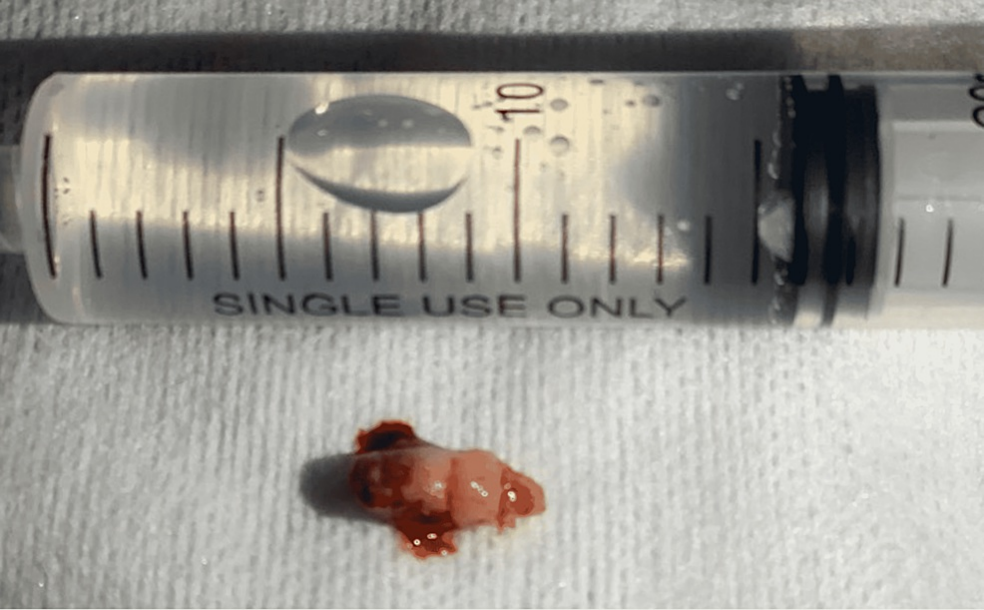
Postoperative picture of the intranasal ectopic tooth next to a syringe.

No irregularities of the permanent dentition were found upon dental examination; hence, the structure was diagnosed as an intranasal ectopic supernumerary tooth. The patient underwent endoscopic intranasal removal of the tooth from the right nostril, performed by an otorhinolaryngology surgeon. The surgery was performed under general anesthesia. After careful inspection of the nasal cavity, the tooth was extracted using Blakesley forceps (Figure 5). Bipolar electrocautery was used for hemostasis. During follow-up examinations at three, six, and 12 months, the patient showed a significant reduction of symptoms with remaining rare reoccurrence of mild sinusitis, which, being more prominent on the contralateral side, most likely had no relation to the ectopic intranasal tooth.

## Discussion

Ectopic teeth can be supernumerary, deciduous or permanent, and can occur in a wide variety of locations outside of the cavity of the mouth, such as the hard palate, maxillary antrum, mandibular condyle, orbit, coronoid process, and nasal cavity. Supernumerary teeth are a rare occurrence that affects only 0.1-1% of the general population, and the incident of such teeth is especially uncommon in the nasal cavity. Supernumerary teeth tend to grow intranasally more often than deciduous or permanent teeth [1-3]. Male predominance (60% of all cases) has been reported in the literature and around half of all patients are diagnosed before adulthood [4,5]. Most commonly, ectopic teeth in the nasal cavity are seen unilaterally, not as multiple teeth in both nasal cavities [4]. It is unknown what determines the development of an intranasal ectopic tooth. A number of potential etiological causes have been noted in the reported cases, including maxillofacial trauma, maxillary osteomyelitis, a developmental defect such as cleft palate, previous odontogenic or rhinogenic infections, exposure to radiation, and hereditary factors including Gardner’s syndrome and cleidocranial dysostosis [4]. Intranasal ectopic teeth occur more often in children with cleft lip and alveolus [4]. However, similar to our case, there is no clear etiological factor in most published cases. There are two theories of the development of supernumerary teeth. According to one theory, supernumerary teeth develop either from a third tooth bed emerging from the dental lamina near the permanent tooth bud or splitting of the permanent bud itself. Another hypothesis states that their development is a reversion to the dentition of extinct primates, which had three pairs of incisors [6,7].

A variety of clinical manifestations are associated with intranasal teeth, such as pain in the face, nose area, difficulty breathing through the nose, foul smell, headache, mild fever, recurrent epistaxis, crusting of the nasal mucosa, localized ulcerations, external deviation of the nose, nasal septal abscess, and nasal-oral fistula. Our patient presented with clear unilateral right-sided symptoms, whereas in the previously reported cases left side involvement was two times more likely [5]. Intranasal teeth may not cause any symptoms at all and can be identified during routine clinical or radiographic examination. [3,5,8,9].

Clinical and radiographic techniques are used for the diagnosis of ectopic intranasal teeth. An intranasal tooth is usually located on the floor of the nasal cavity and can be visualized through an anterior rhinoscopy or endoscopy of the nose. Erupted intranasal teeth may have an appearance of hard white masses embedded in the nasal mucosa, for which diagnosis is usually uncomplicated. [3,5,8]. Similar findings were observed in our case, where the arising of the crown of the tooth from the nasal mucosa was clearly visible during nasal endoscopy. However, in some cases, nasal mucosa may fully cover the tooth and be a source of an underlying infection. This can result in the appearance of necrotic debris, ulcerations, and necrotic tissue. Differential diagnosis should include a foreign body, tumor, rhinolith, osteoma odontoma, exostosis, bony sequestrum, or cyst lesion [3-5,10]. Water’s or Caldwell’s traditional views, a lateral skull view, or panoramic radiography may be helpful diagnostic and management tools for an intranasal tooth that appears as a radiopaque mass with the same radiodensity as that of a tooth [3]. Tooth-equivalent attenuation and centrally located cavity seen as a radiolucent slit or spot in computed tomography (CT) are very distinctive features that help confirm the diagnosis. Moreover, CT findings facilitate surgical planning by enabling the evaluation of the depth of the eruption site, differentiating its situation and anatomical relationship with the other structures [10,11]. With respect to confirmation of diagnosis and further surgical planning, our patient underwent additional CT evaluation after an endoscopic examination.

Surgery is the main treatment option aimed to alleviate symptoms and prevent complications, such as osteomyelitis, nasal septal abscess or perforation, rhinosinusitis, dacryocystitis, oronasal or intraoral fistula, aspergillosis, and nasal deformity [4,7]. The surgeon may suggest either an endoscopic endonasal, a conventional endonasal, or a transoral approach depending on their experience in the endoscopic nasal surgery, patient’s age, presence of a bony socket, and depth of eruption [12]. As with our patient, after multiple consultations with an oral surgeon and otorhinolaryngologist with regard to surgical extraction, the intraoral approach was dismissed and the endoscopic removal of the tooth was chosen as the most conservative surgical method. Endoscopic treatment provides excellent lighting, shorter time of surgery, better visualization, and precise dissection [8,13,14]. If the endoscopic technique fails, the conventional surgical approach can be used [3]. However, according to some publications, regular radiographic and clinical follow-up can be sufficient for asymptomatic patients, and extraction should only be considered after the complete formation of the permanent teeth root, thus minimizing the risk of iatrogenic injury, which can disrupt the development of the dentition [14-16].

## Conclusions

A supernumerary ectopic tooth is a rare phenomenon by itself, yet we report a case where the patient who previously had an ectopic supernumerary tooth in the hard palate presented with a second supernumerary ectopic tooth located intranasally more than a decade later without any clear etiology. Early diagnosis of aberrant teeth can be achieved by clinical and radiological investigations. An endoscopic extraction, which is a favorable way of treatment, is important to prevent potential morbidities and complications associated with foreign objects in the nasal cavity.

## References

[1] Mathur S, Verma B, Dabholkar Y, Saberwal A (2021). Supernumerary tooth in the nasal cavity. J Oral Maxillofac Pathol.

[2] Bergamaschi IP, Olsson B, Sebastiani AM (2019). Intranasal ectopic tooth in adult and pediatric patients: a report of two cases. Case Rep Surg.

[3] Kumar V, Bhaskar A, Kapoor R, Malik P (2020). Conservative surgical management of a supernumerary tooth in the nasal cavity. BMJ Case Rep.

[4] AlMulhim A, AlMomen A, AlKhatib A (2019). Ectopic intranasal canine tooth in a child: a rare case report and literature review. Int J Surg Case Rep.

[5] Lee FP (2001). Endoscopic extraction of an intranasal tooth: a review of 13 Cases. Laryngoscope.

[6] Indeewar H, Dutt SN (2019). Endoscopic removal of intranasal supernumerary tooth: a case report. Indian J Otolaryngol Head Neck Surg.

[7] Anand R, Kieu A, Arulraj E (2021). A rare case of an intra-nasal ectopic tooth in a young woman. Cureus.

[8] Iwai T, Aoki N, Yamashita Y (2012). Endoscopic removal of bilateral supernumerary intranasal teeth. J Oral Maxillofac Surg.

[9] Gupta YK, Shah N (2001). Intranasal tooth as a complication of cleft lip and alveolus in a four year old child: case report and literature review. Int J Paediatr Dent.

[10] Min HJ, Oh SR, Kim KS (2020). Endoscopic differences between intranasal ectopic teeth. Ear Nose Throat J.

[11] Al Dhafeeri HO, Kavarodi A, Al Shaikh K, Bukhari A, Al Hussain O, El Baramawy A (2014). Recurrent epistaxis caused by an intranasal supernumerary tooth in a young adult. Am J Case Rep.

[12] Dokania V, Kinnera H, S S, Shetty N, Gaikwad N (2021). Ectopic deciduous maxillary tooth in the nasal cavity following trauma. Cureus.

[13] Yu C, Gu D, An J, Tang Y (2015). Case presentation of an intranasal ectopic tooth in a pediatric patient. Am J Otolaryngol.

[14] Kim DH, Kim JM, Chae SW, Hwang SJ, Lee SH, Lee HM (2003). Endoscopic removal of an intranasal ectopic tooth. Int J Pediatr Otorhinolaryngol.

[15] Agrawal M, Khan TS, Gupta T, Khanna S (2014). Intranasal tooth: ectopic eruption 1 year after maxillofacial trauma. BMJ Case Rep.

[16] Choi YS, Kim YD, Bae CH, Na HG (2021). Intranasal supernumerary tooth in a child: a case report. Turk J Pediatr.

